# Prognostic Value of the LATITUDE and CHAARTED Risk Criteria for Predicting the Survival of Men with Bone Metastatic Hormone-Naïve Prostate Cancer Treated with Combined Androgen Blockade Therapy: Real-World Data from a Japanese Multi-Institutional Study

**DOI:** 10.1155/2020/7804932

**Published:** 2020-07-01

**Authors:** Takashi Kawahara, Shuko Yoneyama, Yoshio Ohno, Junpei Iizuka, Yasunobu Hashimoto, Hideyasu Tsumura, Ken-ichi Tabata, Yoshihiro Nakagami, Kazunari Tanabe, Masatsugu Iwamura, Hiroji Uemura, Yasuhide Miyoshi

**Affiliations:** ^1^Departments of Urology and Renal Transplantation, Yokohama City University Medical Center, Yokohama, Japan; ^2^Department of Urology, Tokyo Medical University, Tokyo, Japan; ^3^Department of Urology, Tokyo Women's Medical University, Japan; ^4^Department of Urology, Saiseikai Kawaguchi General Hospital, Saitama, Japan; ^5^Department of Urology, Kitasato University School of Medicine, Sagamihara, Japan

## Abstract

**Background:**

The CHAARTED and LATITUDE trials demonstrated a prolonged overall survival (OS) for metastatic hormone-naïve prostate cancer (mHNPC) patients who receive up-front docetaxel or abiraterone acetate. These studies used their own risk criteria: CHAARTED trial defines high- and low-volume diseases and LATITUDE trial targeting a high-risk disease. The present study explored whether or not the CHAARTED and LATITUDE criteria were useful for predicting the outcome in Japanese bone mHNPC patients, including elderly patients (≥70 years).

**Methods:**

A total of 532 mHNPC patients diagnosed from 2004 to 2014 in multithird referral cancer centers were enrolled in this study. All patients had bone metastasis and received combined androgen blockade treatment as an initial hormonal therapy.

**Results:**

The number of patients with CHAARTED low-volume and high-volume diseases was 178 (33.5%) and 354 (66.5%), respectively. On the contrary, the number of patients with LATITUDE low-risk and high-risk diseases was 157 (29.5%) and 375 (70.5%), respectively. A total of 307 (57.7%) patients were defined as having both CHAARTED high-volume and LATITUDE high-risk disease. The median castration-resistant prostate cancer- (CRPC-) free survival was 12.5 months for the CHAARTED high volume, 56.9 months for the CHAARTED low volume, 13.6 months for the LATITUDE high risk, and 37.3 months for the LATITUDE low risk, respectively. The OS was 50.1 months in patients with CHAARTED high-volume disease, 95.1 months in patients with CHAARTED low-volume disease, 54.0 months in patients with LATITUDE high-risk disease, and 92.7 months in patients with LATITUDE low-risk disease, respectively. This trend was also observed in elderly (≥70 years old) patients.

**Conclusions:**

The patients with CHAARTED high-volume disease or LATITUDE high-risk disease showed a shorter CRPC-free survival and a shorter OS than those in the CHAARTED low-volume disease group or in the LATITUDE low-risk group among Asian Japanese bone metastatic HNPC patients.

## 1. Introduction

With the recent spread of prostate-specific antigen (PSA) health checks, the early-stage diagnosis of prostate cancer is becoming increasingly frequent. However, a substantial number of patients are still diagnosed with metastatic prostate cancer [1, 2]. In the past, androgen deprivation therapy (ADT) was considered to be the standard treatment for metastatic hormone-naïve prostate cancer (mHNPC). Recently, the CHAARTED and LATITUDE trials revealed a prolonged overall survival (OS) for mHNPC patients using up-front docetaxel (DOC) and abiraterone acetate (ABI) [3, 4].

These two studies independently set risk criteria; the CHARTTED trial used high- and low-volume criteria, while the LATITUDE trial used high- and low-risk criteria. LATITUDE low risk was set as the non-high-risk LATITUDE criteria. The CHAARTED trial revealed that up-front DOC systemic chemotherapy combined with ADT prolonged the OS compared with ADT monotherapy [4]. A subgroup analysis showed that the high-volume group obtained a longer OS, while the low-volume group did not. The LATITUDE trial reveled that ABI and prednisolone (PSL) combined with ADT prolonged the OS compared with ADT monotherapy among high-risk mHNPC patients [3].

Some studies have found that ADT was more effective in Asian populations, including Japanese, than in western ones [5]. The Japanese population routinely ranks at the top for global life expectancy, and prostate cancer is often diagnosed at an advanced stage in elderly men [6]. Due to the evidence of the LATITUDE and CHAARTED studies, abiraterone acetate and DOC would be more frequently introduced in the treatment of mHNPC patients. However, these risk criteria were only confirmed in patients in a clinical trial registry. These risk criteria as risk categories have not yet been confirmed in real-world settings.

The present study explored whether or not the CHAARTED and LATITUDE criteria were also prognostic risk factors in Japanese bone mHNPC patients, including elderly patients.

## 2. Materials and Methods

### 2.1. Patients

A total of 532 mHNPC patients diagnosed from 2004 to 2014 in multithird referral cancer centers were enrolled in this study. All patients had bone metastasis and received combined androgen blockade (CAB) using LHRH analogue or agonist combination with 80 mg of bicalutamide treatment as an initial hormonal therapy.

The institutional review board of Yokohama City University Medical Center approved this study (B180500019). The definition of castration-resistant prostate cancer (CRPC) was set by the Prostate Cancer Working Group 2 [7].

To determine the utility of the CHAARTED and LATITUDE risk criteria as risk factors in Japanese patients, we first checked whether the era of the initial prostate cancer diagnosis affected the prognosis for OS and CRPC-free survival, respectively. We then compared the prognosis between CHAARTED criteria with a high volume and that with a low volume. CHAARTED high-volume disease was defined as the presence of visceral metastasis or ≥4 bone lesions with ≥1 beyond the vertebral bodies and pelvis. LATITUDE high risk was defined as meeting at least two of the following three criteria: (i) Gleason score ≥ 8, (ii) presence of ≥3 lesions on bone scan, and (iii) presence of measurable visceral lesions (Supplementary Table 1). We also compared the prognosis between the LATITUDE criteria with a high risk and that with a low risk.

We then observed that CHAARTED criteria high volume and LATITUDE criteria high risk also had a poorer CRPC-free survival than the low-volume and low-risk group in Japanese bone mHNPC patients who had undergone CAB treatment. We also compared the association of a CHAARTED high volume and a LATITUDE high risk with the prognosis to that between a CHAARTED low volume and a LATITUDE low risk and the prognosis in Japanese bone mHNPC patients who had undergone CAB treatment. We also determined the utility of these risk criteria in elderly patients (≥70 years old).

### 2.2. Statistical Analyses

The patients' characteristics and preoperative factors were analyzed by the Mann-Whitney *U* and chi-square tests, using the GraphPad Prism software program (GraphPad Software, La Jolla, CA, USA). The survival duration was defined as the time between the dates of initial hormonal therapy installation and the time of CRPC or death. Univariable and multivariable analyses were conducted using the Cox proportional hazard model with stepwise regression analyses. We first performed a univariable analysis including the risk factors of age, Gleason score, PSA, Hb, LDH, ALP, and visceral metastasis in addition to the LATITUDE and CHAARTED criteria, and then, we performed a multivariable analysis including the risk factors shown to significantly influence the OS in the univariate analysis. A log-rank test was performed for comparisons between higher and lower risk according to the CHAARTED and LATITUDE criteria. *p* values of <0.05 were considered to indicate statistical significance.

## 3. Results

### 3.1. Patients' Characteristics

**Figure 1:**
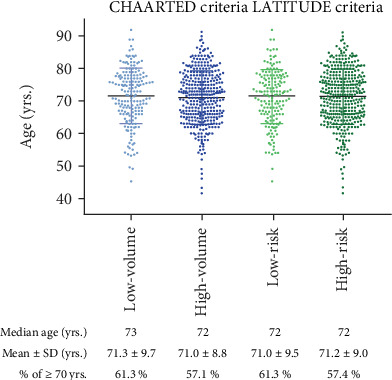
Patients' age distribution in LATITUDE and CHAARTED risk criteria.

A total of 532 patients were enrolled in this study. Fifty-two (9.8%) cases had visceral metastasis. The clinical characteristics, including the Gleason score, PSA, hemoglobin, and LDH, are summarized in Table 1. The median observation period was 44.0 months. There were no significant differences in the CRPC-free survival and OS among the patients diagnosed 2004-2006, 2007-2012, and 2013-2015 (Supplementary Fig. 1). The median (mean ± standard deviation (SD)) age was 72.0 (71.4 ± 8.3) years old. The median (mean ± SD) age for each risk criteria set was 73.0 (71.7 ± 8.6) years old for the CHAARTED low volume, 72.0 (71.2 ± 8.2) years old for the CHAARTED high volume, 72.0 (71.4 ± 8.2) years old for the LATITUDE low risk, and 72.0 (71.4 ± 8.4) years old for the LATITUDE high risk, respectively (Figure 1). There were no marked age differences among the risk groups. The percentage of cases that were 70 years of age or older in each group was 61.3% for a low volume, 57.1% for a high volume, 61.3% for a low risk, and 57.4% for a high risk.

### 3.2. Distribution of the CHAARTED and LATITUDE Risk Criteria

**Figure 2:**
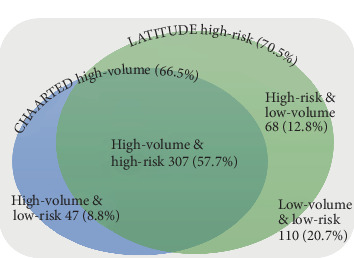
Patients' distribution according to LATITUDE and CHAARTED risk criteria for bone metastatic prostate cancer.

According to the CHAARTED criteria, 178 (33.5%) had a low volume, and 354 (66.5%) had a high volume. According to the LATITUDE criteria, 157 (29.5%) had a low risk, and 375 (70.5%) had a high risk. A total of 307 (57.7%) patients were classified as having both a CHAARTED high volume and a LATITUDE high risk (Figure 2).

### 3.3. The Prognosis according to the CHAARTED and LATITUDE Risk Criteria

**Figure 3:**
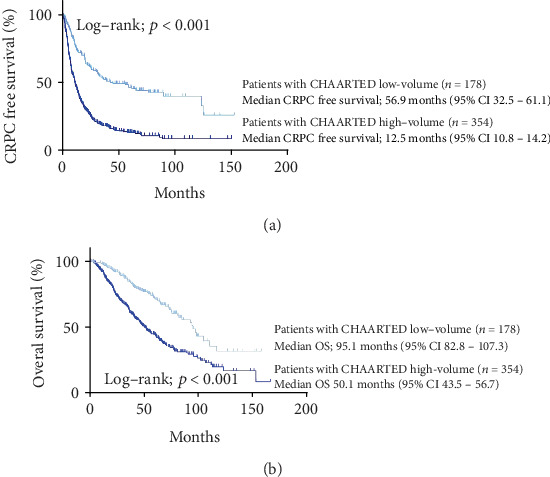
(a) CRPC-free and (b) overall survival according to the CHAARTED risk criteria.

**Figure 4:**
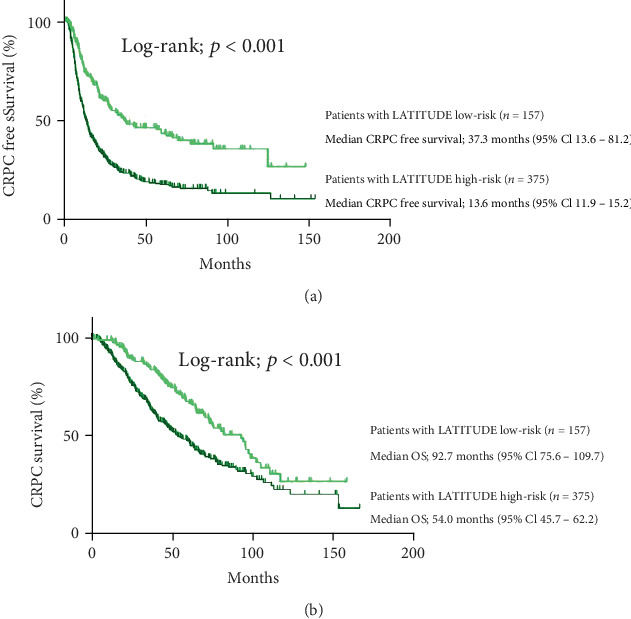
(a) CRPC-free and (b) overall survival according to the LATITUDE risk criteria.

A summary of the CRPC-free survival and OS in the CHAARTED study (high volume, low volume, and all patients), the LATITUDE study (high risk and Japanese cohort), and the current study (high volume, low volume, high risk, and low risk cohort) is shown in Table 2. The median CRPC-free survival in our cohort was 12.5 mo. as defined with CHAARTED-high and 56.9 mo. defined with CHAARTED-low, respectively. When we compared our cohort with the CHAARTED-ADT alone arm, our cohort with CHAARTED-high arm had a 3.9 mo. longer median CRPC-free survival than the CHAARTED-high-ADT alone arm. Our cohort with a CHAARTED-low arm had a 34.2 mo. longer median CRPC-free survival than CHAARTED-low ADT alone arm. The median CRPC-free survival in our cohort was 13.6 mo. as defined with a LATITUDE-high arm and 37.3 mo. as defined with a LATITUDE-low arm, respectively. When we compared our cohort with the LATITUDE-high-ADT alone arm, our LATITUDE-high arm had a 6.2 mo. longer median CRPC-free survival. A subgroup analysis of the original LATITUDE study in a Japanese cohort subanalysis showed that the CRPC-free survival was 9.26 months [8]. The patients defined as both the CHAARTED high-volume group and the LATITUDE high-risk group showed a significantly poorer CRPC-free survival in our cohorts than for the low-volume and low-risk groups (*p* < 0.001 and *p* < 0.001, respectively) (Table 2, Figures 3 and 4). We also confirmed the differences according to the number of sites of bone metastasis. The group with ≥3 sites of metastasis showed a significantly poorer OS in comparison to the group with <3 sites of metastasis (Supplementary Figure 2).

The OS in ADT treatment in our cohort was 50.1 months for the CHAARTED high-volume group and 95.1 months for the CHAARTED low-volume group and 54.0 months for the LATITUDE high-risk group and 92.7 months for the LATITUDE low-risk group. Compared to the CHAARTED study cohort, the OS in our cohort was 17.9 months longer for the high-volume group. The high-risk group in this cohort had a survival of 17.5 months longer than the LATITUDE study criteria which had a survival of 36.5 months. In our cohort, both the CHAARTED high-volume criteria and the LATITUDE high risk criteria showed a significantly poorer OS in our cohorts than in the low-volume and low-risk group (*p* < 0.001 and *p* < 0.001, respectively) (Table 2, Figures 3 and 4). The multivariable analysis revealed that the CHAARTED high-volume criteria and the LATITUDE high-risk criteria were independent risk factors for a poor prognosis (hazard ratio (HR): 1.73, 95% confidence interval (CI): 1.21-2.50, *p* = 0.003 in the CHAARTED high-volume criteria; HR: 1.50, 95% CI: 1.09-2.06, *p* = 0.014 in the LATITUDE high-risk criteria) (Table 3).

### 3.4. The Prognosis according to the CHAARTED and LATITUDE Risk Criteria for Elderly Patients

Among elderly mHNPC patients, those in the CHAARTED high-volume group and LATITUDE high-risk group showed both a poorer CRPC-free survival and OS than the others. The CHAARTED high-volume group showed a significantly poorer CRPC-free survival than the low-volume group (66.3 vs. 14.7 months, *p* < 0.001), and the LATITUDE high-risk group also showed a significantly poorer CRPC-free survival than the low-risk group (36.4 vs. 14.2 months, *p* < 0.001). The CHAARTED high-volume group showed a significantly poorer OS than the low-volume group (92.7 vs. 50.6 months, *p* = 0.006), and the LATITUDE high-risk group also showed a significantly poorer OS than the low-risk group (82.1 vs. 50.1 months, *p* = 0.001) (Supplementary Figures 3 and 4).

## 4. Discussion

This study showed the CHAARTED risk criteria and LATITUDE risk criteria which were adapted to Japanese mHNPC patients. These criteria are useful as prognostic factors in Japanese mHNPC patients regardless of age. These risk criteria were also useful as a prognostic factor for elderly patients (>70 years of age). These findings indicated that these criteria would be useful in patients who are typically excluded from clinical trials. Therefore, when considering initial therapies using DOC and ABI for Japanese mHNPC patients, the CHAARTED and LATITUDE risk criteria may be useful for selecting candidate mHNPC patients for up-front DOC treatment.

In this cohort, the CHAARTED high-volume group accounted for 66.5% (354/532), and LATITUDE high-risk group accounted for 70.5% (375/532) of all bone metastatic prostate cancer patients. Okamoto et al. similarly found that the CHAARTED high-volume group accounted for 63%, while the LATITUDE high-risk group accounted for 59% of Japanese mHNPC patients in their study [2]. The differences in the LATITUDE criteria might be influenced by the fact that the present study included only bone metastatic cases. Most patients who satisfied at least one of the risk criteria set showed both the CHAARTED high-volume and LATITUDE high-risk group to comprise 72.7% (307/422), while the remaining 27.3% (115/422) had one of the risk criteria.

Several previous studies have reported a higher response rate of ADT in Asian patients than in western patients, especially those of African and Caucasian descent [1, 9]. In the present study, the CRPC-free survival was 56.9 months in the CHAARTED low-volume group, 12.5 months in the CHAARTED high-volume group, 37.3 months in the LATITUDE low-risk group, and 13.6 months in the LATITUDE high-risk group. The CHAARTED study revealed a CRPC-free survival of 22.7 months in the low-volume ADT group and 8.6 months in the high-volume ADT group, while the LATITUDE study revealed a CRPC-free survival of 7.4 months in the high-risk ADT group. These real-world Japanese data indicated a favorable response to initial ADT treatment. The median OS in our cohort with the LATITUDE high-risk group was 54.0 mo. In the LATITUDE study in a Japanese cohort, OS was not reached in both the abiraterone acetate and placebo groups and the 5-year survival rates were 69.2% in the abiraterone acetate group and 53.7% in the placebo group with a median follow-up of 56.6 months [10]. The LATITUDE study included Japanese patients and reported efficacy in the Japanese cohort, while the CHAARTED study included no Japanese patients. Further study is therefore needed in order to confirm the efficacy of up-front DOC treatment in Japanese mHNPC patients [8].

Several limitations associated with the present study warrant mention. This study retrospectively evaluated the risk criteria used in the CHAARTED and LATITUDE trials in Japanese bone metastatic HNPC patients. The present cohort did not include up-front treatment, so the response of up-front treatments was not evaluated. A second limitation is that we included patients diagnosed from 2004 to 2014. Under the Japanese insurance system, enzalutamide and abiraterone were introduced for clinical use in 2014; before that point, DOC had been the only established treatment for CRPC. Differences in the available treatment options might therefore have affected the outcomes. On the other hand, this study showed no differences in either the CRPC-free survival or OS regarding the diagnostic period. This study included 101 patients (18.9%) who received initial radiation therapy. We also suspected that the time from the initial diagnosis and CRPC differed in each of the prostate cancer patients. Thus, a larger study is needed to confirm the efficacy of newly emerging drugs for CRPC.

## 5. Conclusion

The CHAARTED high-volume and LATITUDE high-risk group showed a shorter CRPC-free survival and a poorer OS than the CHAARTED low-volume group and the LATITUDE low-risk group among Japanese bone metastatic prostate cancer patients. This trend was also observed in elderly (≥70 years old) patients. The CHAARTED high-volume and LATITUDE high-risk criteria are therefore reasonable risk factors for use in Japanese mHNPC patients.

## Figures and Tables

**Table 1:** Patients' characteristics.

Variables	Entire cohort (*n* = 532)	Patients with LATITUDE high risk (*n* = 375)	Patients with LATITUDE low risk (*n* = 157)	Patients with CHAARTED high-volume (*n* = 354)	Patients with CHAARTED low-volume (*n* = 178)
Median age (years) (range)	72 (42-92)	72 (42-91)	72 (45-92)	72 (42-91)	73 (45-92)
No. of patients with LATITUDE high risk, *n* (%)	375 (70.5)	375 (100.0)	0 (0.0)	307 (86.7)	68 (38.2)
No. of patients with high-volume disease defined by CHAARTED, *n* (%)	354 (66.5)	307 (81.9)	47 (29.9)	354 (100.0)	0 (0.0)
No. of patients with visceral metastases, *n* (%)	52 (9.8)	44 (11.7)	8 (5.0)	47 (13.3)	0 (0.0%)
No. of patients with ≥3 bone mets	405 (76.1)	350 (93.3)	55 (35.0)	341 (96.3)	64 (12.0%)
No. of patients with Gleason score ≥ 8	443 (83.3)	373 (99.5)	70 (44.6)	306 (86.4)	137 (25.8%)
PSA (ng/mL) (range)	280.4 (0.7-25000.0)	349.0 (0.7-25000.0)	127.9 (4.0-17615.0)	457.2 (0.7-23474.0)	84.6 (4.0-25000.0)
Hemoglobin (g/dL) (range)	13.2 (6.5-17.3)	13.0 (6.5-16.9)	13.6 (7.3-17.3)	13.0 (6.5-16.6)	13.7 (7.3-17.3)
LDH (IU/L) (range)	203 (75-3025)	210 (75-3025)	194 (90-419)	215 (118-3025)	188 (75-416)
ALP (IU/L) (range)	397 (113-16050)	515 (115-16050)	280 (113-3970)	570 (115-416050)	262 (113-2526)

PSA: prostate-specific antigen; LDH: lactate dehydrogenase; ALP: alkaline phosphatase.

**Table 2:** CRPC-free and overall survival in each study.

CRPC-free survival	Low volume	High volume	OS	Low volume	High volume
CHAARTED	31.0/22.7	14.9/8.6	CHAARTED	NR/NR	49.2/32.2
CHAARTED (all patients)	20.2/11.7		CHAARTED (all patients)	57.6/44.0	
Our cohort	56.9	12.5	Our cohort	95.1	50.1

CRPC-free survival	Low risk	High risk	OS	Low risk	High risk
LATITUDE	—	33.2/7.4	LATITUDE	—	53.3/36.5
LATITUDE (Japanese cohort)	—	NR/9.3	LATITUDE (Japanese cohort)	—	NR/NR
Our cohort	37.3	13.6	Our cohort	92.7	54.0

**Table 3:** Univariable and multivariable analyses of factors associated with the overall survival.

Variables	Univariable				Multivariable				Multivariable			
	*p* value	HR	95% CI		*p* value	HR	95% CI		*p* value	HR	95% CI	
			Lower	Upper			Lower	Upper			Lower	Upper
Age ≥ 72 vs. <72 (years)	0.221	1.16	0.91	1.47								
Gleason score ≥ 8 vs. ≤7	0.164	1.27	0.91	1.77								
Visceral metastasis	0.766	0.94	0.62	1.42								
PSA ≥ 280.4 vs. <280.4 (ng/mL)	0.001	1.48	1.16	1.88	0.252	0.84	0.62	1.13	0.605	0.93	0.69	1.24
Hb < 13.2 vs. ≥13.2 (g/dL)	<0.001	2.44	1.88	3.16	<0.001	2.16	1.63	2.88	<0.001	2.11	1.59	2.79
LDH ≥ 203 vs. <203 (IU/L)	<0.001	2.27	1.77	2.90	<0.001	2.00	1.50	2.67	<0.001	2.08	1.56	2.77
ALP ≥ 397 vs. <397 (IU/L)	<0.001	1.91	1.49	2.46	0.091	1.30	0.96	1.75	1.413	1.06	1.89	
CHAARTED high volume vs. low volume	<0.001	2.21	1.66	2.93	0.003	1.73	1.21	2.50	—	—	—	—
LATITUDE high risk vs. low risk	<0.001	1.72	1.31	2.27	—	—	—	—	0.014	1.50	1.09	2.06

PSA: prostate-specific antigen; Hb: hemoglobin; LDH: lactate dehydrogenase; ALP: alkaline phosphatase.

## Data Availability

The data used to support the findings of this study are available from the corresponding author upon request.

## Supplementary Materials

Supplementary Materials Fig. 1: (a) CRPC-free and (b) overall survival in each diagnostic era. Supplementary Fig. 2: overall survival in patients with ≥3 vs. <3 sites of bone metastasis. Supplementary Fig. 3: overall survival in (a) less than 70 yrs. and (b) 70 yrs. or older with the CHAARTED risk criteria. Overall survival in (c) less than 70 yrs. and (d) 70 yrs. or older with the LATITUDE risk criteria. Supplementary Fig. 4: CRPC-free survival in (a) less than 70 yrs. and (b) 70 yrs. or more in CHAARTED risk criteria. CRPC-free survival in (c) less than 70 yrs. and (d) 70 yrs. or more in LATITUDE risk criteria. Supplementary Table 1: CHAARTED high volume and LATITUDE high risk.
