# Supplementary material for: Prognostic Value of the LATITUDE and CHAARTED Risk Criteria for Predicting the Survival of Men with Bone Metastatic Hormone-Naïve Prostate Cancer Treated with Combined Androgen Blockade Therapy: Real-World Data from a Japanese Multi-Institutional Study
Source: Biomed Res Int. 2020 Jul 1;2020:7804932. doi: 10.1155/2020/7804932 (PMC7352129; doi:10.1155/2020/7804932)
Supplement: Supplementary Materials — Fig. 1: (a) CRPC-free and (b) overall survival in each diagnostic era. Supplementary Fig. 2: overall survival in patients with ≥3 vs. <3 sites of bone metastasis. Supplementary Fig. 3: overall survival in (a) less than 70 yrs. and (b) 70 yrs. or older with the CHAARTED risk criteria. Overall survival in (c) less than 70 yrs. and (d) 70 yrs. or older with the LATITUDE risk criteria. Supplementary Fig. 4: CRPC-free survival in (a) less than 70 yrs. and (b) 70 yrs. or more in CHAARTED risk criteria. CRPC-free survival in (c) less than 70 yrs. and (d) 70 yrs. or more in LATITUDE risk criteria. Supplementary Table 1: CHAARTED high volume and LATITUDE high risk. [file 7804932.f1.pdf]

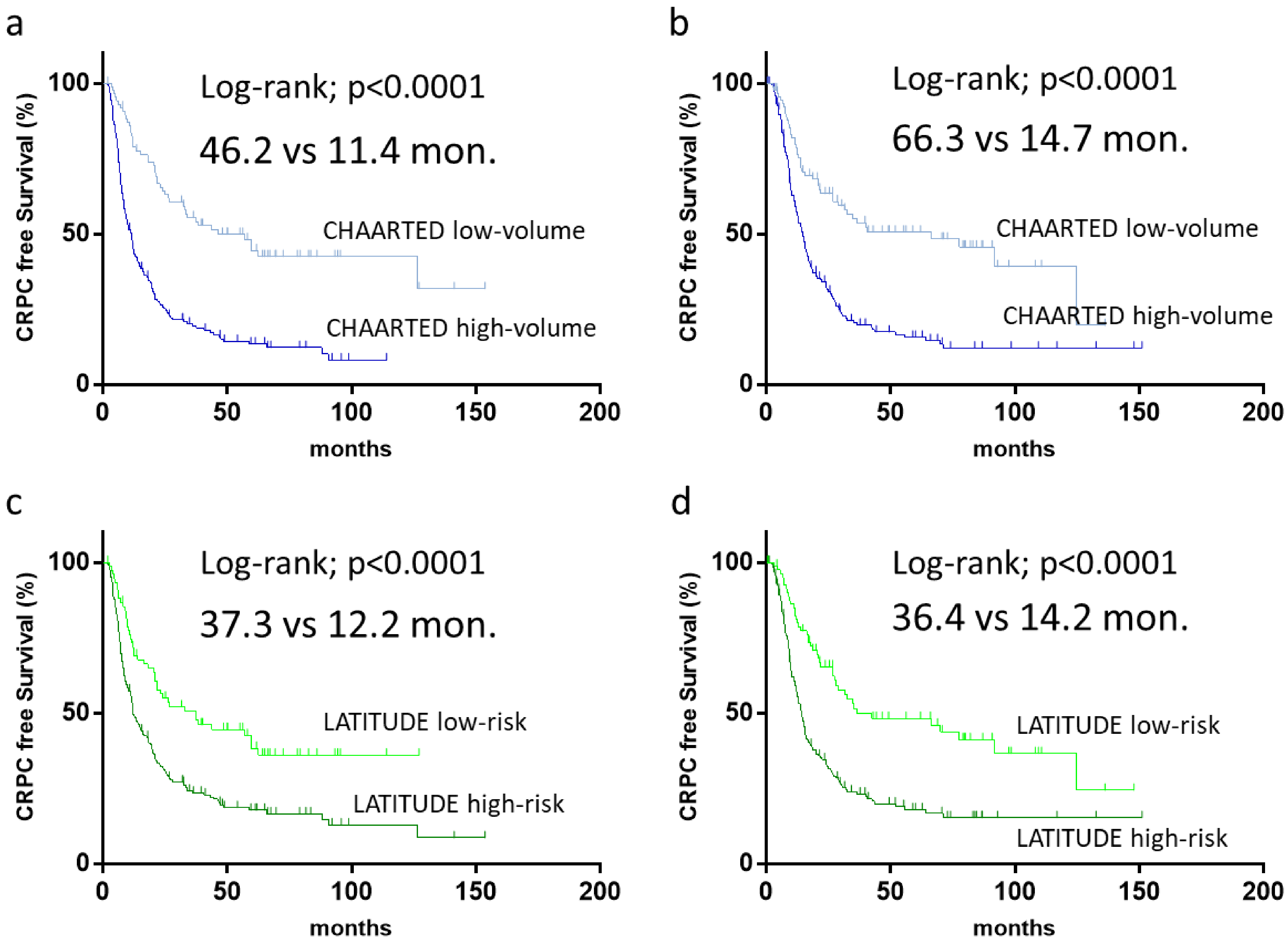

a
Log-rank; p<0.0001
46.2 vs 11.4 mon.
CHAARTED low-volume
CHAARTED high-volume
CRPC free Survival (%)
months
b
Log-rank; p<0.0001
66.3 vs 14.7 mon.
CHAARTED low-volume
CHAARTED high-volume
c
Log-rank; p<0.0001
37.3 vs 12.2 mon.
LATITUDE low-risk
LATITUDE high-risk
d
Log-rank; p<0.0001
36.4 vs 14.2 mon.
LATITUDE low-risk
LATITUDE high-risk

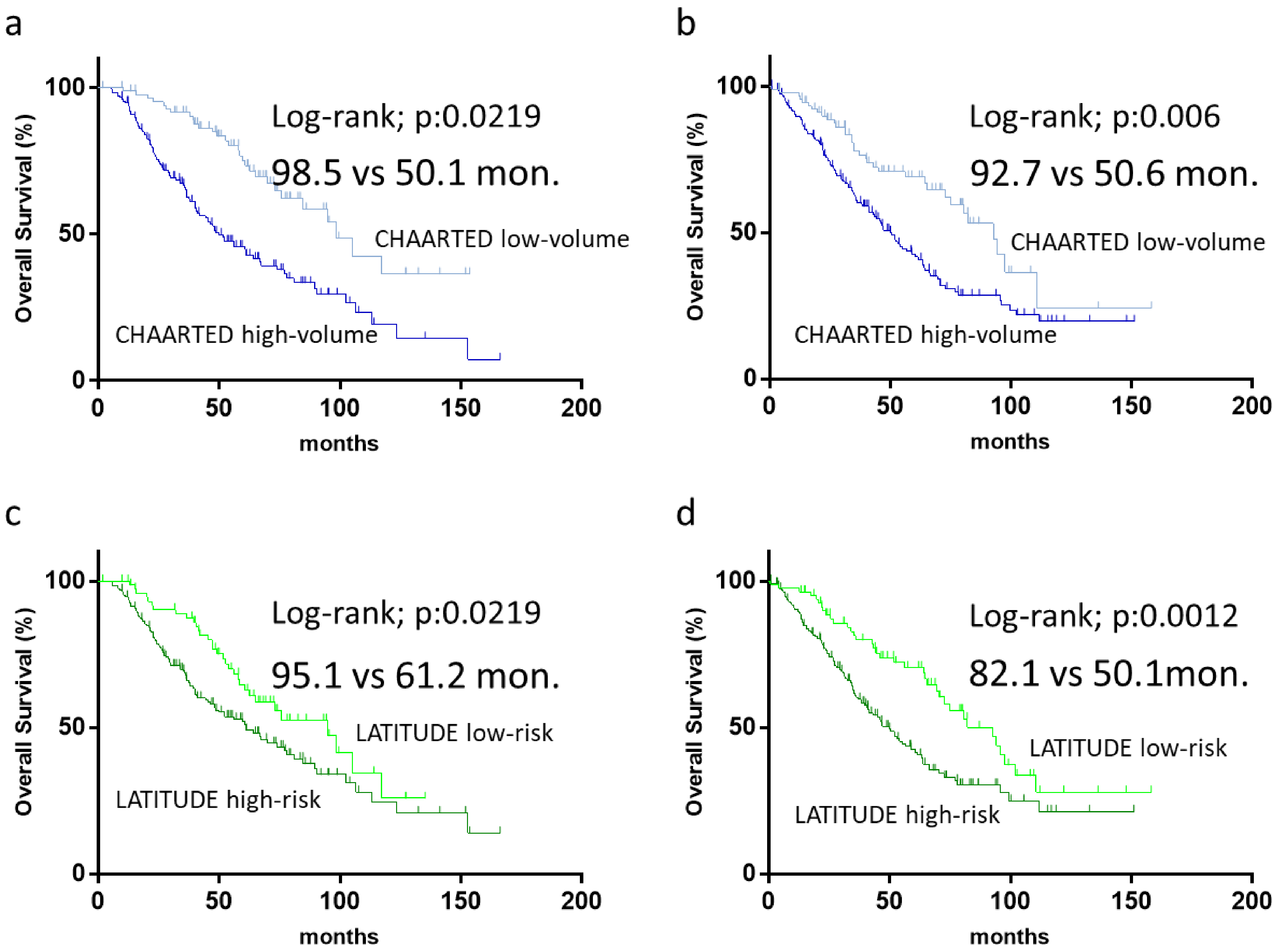
a
Log-rank; p:0.0219
98.5 vs 50.1 mon.
CHAARTED low-volume
CHAARTED high-volume
Overall Survival (%)
months
b
Log-rank; p:0.006
92.7 vs 50.6 mon.
CHAARTED low-volume
CHAARTED high-volume
Overall Survival (%)
months
c
Log-rank; p:0.0219
95.1 vs 61.2 mon.
LATITUDE low-risk
LATITUDE high-risk
Overall Survival (%)
months
d
Log-rank; p:0.0012
82.1 vs 50.1mon.
LATITUDE low-risk
LATITUDE high-risk
Overall Survival (%)
months

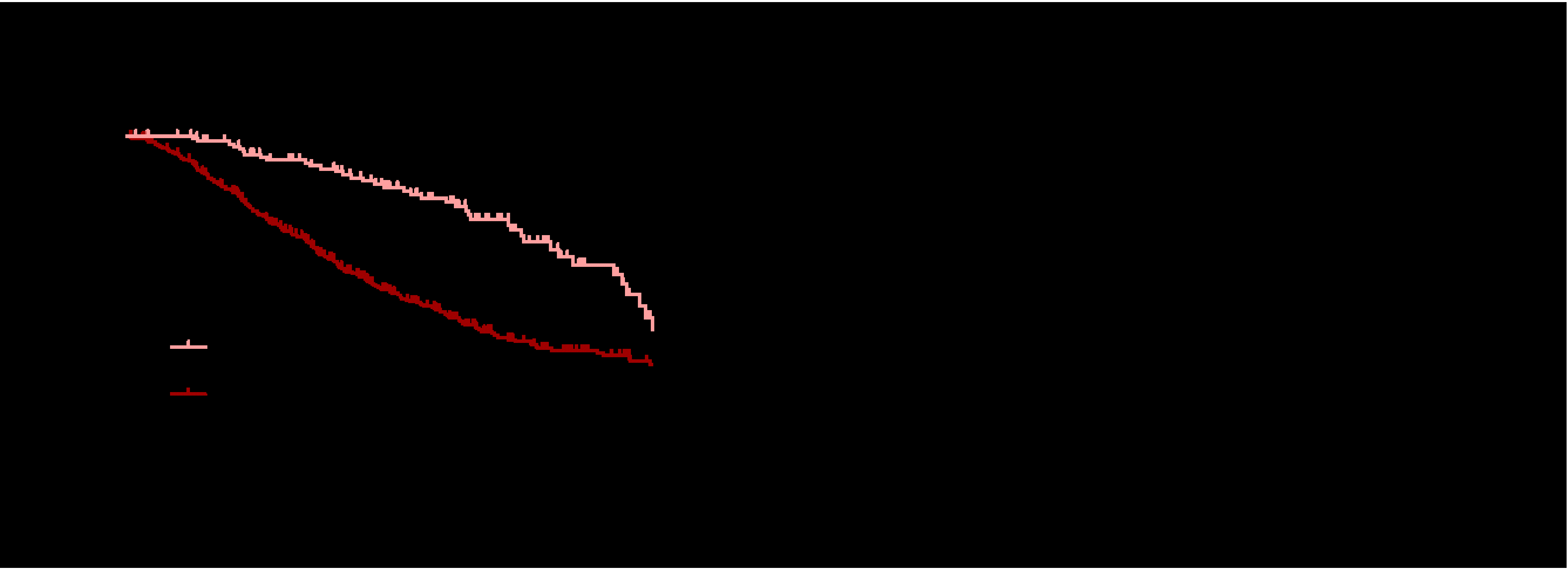

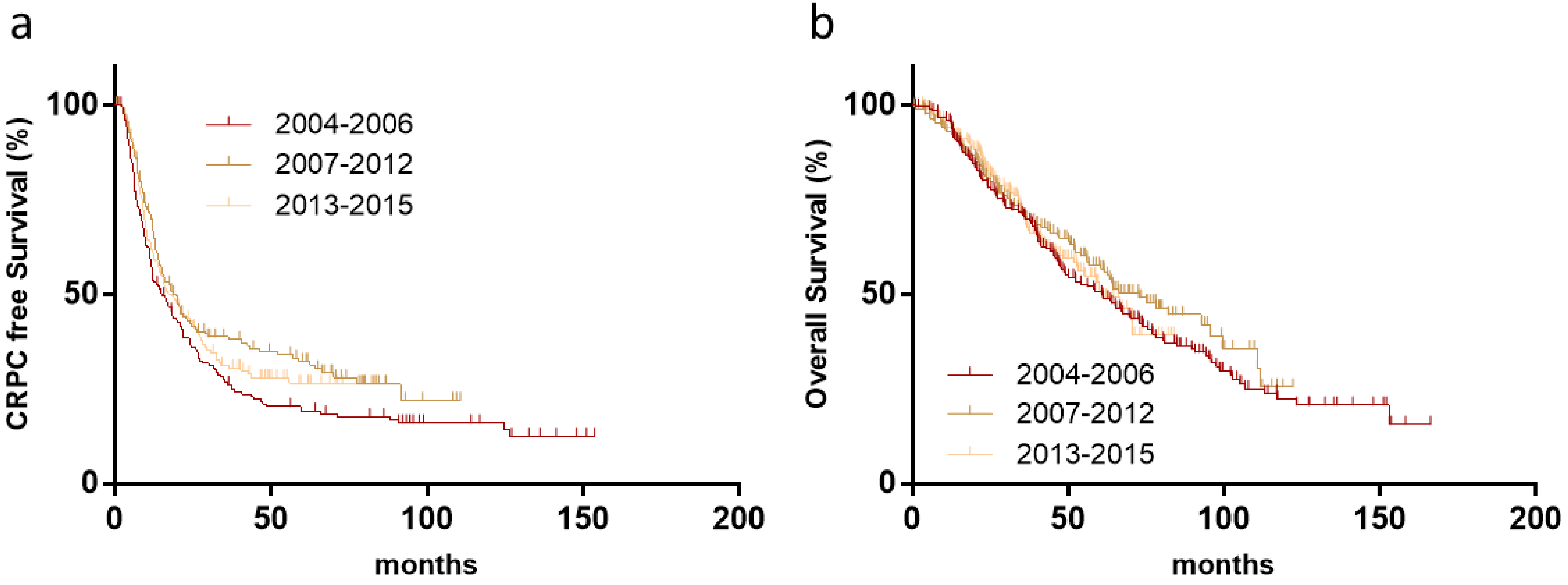

a
CRPC free Survival (%)
100
50
0
2004-2006
2007-2012
2013-2015
0
50
100
150
200
months
b
Overall Survival (%)
100
50
0
2004-2006
2007-2012
2013-2015
0
50
100
150
200
months

Supplementary Table 1

CHAARTED High Volume	LATITUDE High Risk
1. Visceral metastasis	1. Visceral metastasis*
or	2. Bone metastasis (≥3 sites)*
2. Bone metastasis (≥4 sites)*	3. Gleason Score (≥8)*
*At least one spine or extra pelvic	*Two or more
